# Prescribing and dosing patterns of ferric carboxymaltose for iron deficiency anemia: a real-world study from Saudi Arabia

**DOI:** 10.3389/fmed.2026.1864516

**Published:** 2026-08-07

**Authors:** Yahya A. Alzahrani, Ayed A. Alkatheeri, Abdulrahman B. Alshamrani, Abdullah M. Alzahrani, Thamer M. A. Alqurashi, Maan H. Harbi, Rajaa Al-Raddadi

**Affiliations:** 1 Department of Pharmacology, Faculty of Medicine, King Abdulaziz University, Rabigh, Saudi Arabia; 2 Drug Information Center, Pharmacy Department, East Jeddah Hospital, Jeddah First Health Cluster, Jeddah, Saudi Arabia; 3 Clinical Pharmacy Unite, Pharmacy Department, East Jeddah Hospital, Jeddah First Health Cluster, Jeddah, Saudi Arabia; 4 Pharmaceutical Care Department, Ministry of National Guard—Health Affairs, Jeddah, Saudi Arabia; 5 Pharmacology and Toxicology Department, College of Pharmacy, Umm Al-Qura University, Makkah, Saudi Arabia; 6 Department of Preventive Medicine and Public Health, Faculty of Medicine, King Abdulaziz University, Jeddah, Saudi Arabia

**Keywords:** dosing appropriateness, ferric carboxymaltose, iron deficiency anemia, prescribing patterns, Saudi Arabia

## Abstract

**Introduction:**

Ferric carboxymaltose (FCM) is increasingly used for iron deficiency anemia, yet real-world prescribing patterns and dosing appropriateness remain poorly characterized, particularly in regions with high anemia prevalence. This study evaluated FCM prescribing patterns, dosing appropriateness, and associated clinical and financial implications at a Saudi tertiary hospital.

**Methods:**

This retrospective observational study examined adult patients who received FCM at a tertiary care general hospital in Saudi Arabia over a 6-month period. Dosing appropriateness was determined by comparing prescribed cumulative doses (PCD) with calculated cumulative doses (CCD) per the Summary of Product Characteristics. Hemoglobin response and financial impact were also assessed.

**Results:**

Of 182 patients treated with FCM, 79 met the inclusion criteria (97.5% female; mean age 34.6 ± 8.3 years). Overall, 82% of prescriptions exhibited an inappropriate cumulative dose, with 88% subtherapeutic. Mean PCD (1,118 ± 492 mg) was substantially lower than CCD (1,640 ± 438 mg), a mean under-dosing gap of 522.6 mg (*p* < 0.001). In addition, 63.3% of patients were iron-therapy naïve. Hemoglobin response was reduced (1.35 g/dL rise) versus suitably dosed populations (2.5–3.0 g/dL). No predictor of inappropriate dosing reached significance. Projected complication costs (171,055 SAR) substantially exceeded apparent medication savings (19,599 SAR).

**Discussion:**

Widespread subtherapeutic FCM dosing compromises therapeutic outcomes and increases downstream costs. Implementation of electronic clinical decision support and standardized dosing protocols is urgently needed to optimize intravenous iron utilization.

## 1 Introduction

Iron deficiency anemia (IDA) affects roughly 25% of the world’s population and is the most common nutritional deficit worldwide (1, 2). According to the World Health Organization, about 500 million women of reproductive age worldwide suffer from anemia, with iron deficiency being the primary cause. Women are more susceptible to IDA due to menstrual blood loss, nutritional variables, and higher iron requirements during reproductive years (3–5). Untreated IDA can cause fatigue, lower work capacity, poor cognitive function, and diminished quality of life in addition to hematological abnormalities (6).

In Saudi Arabia, the prevalence of IDA remains a significant public health concern, despite economic development and improvements to healthcare facilities. According to national epidemiological surveys, IDA prevalence ranges from 10 to 60% among various demographic groups (7–9). A comprehensive study by Alquaiz et al. discovered that anemia affected roughly 40% of Saudi women of reproductive age in Riyadh, with iron deficiency being the most common cause (10). Dietary habits characterized by low consumption of iron-rich foods, frequent tea and coffee drinking, which restrict iron absorption, and a lack of information about balanced nutrition are all contributing factors in the Saudi context (11–13). These characteristics make the population particularly reliant on efficient iron supplementation measures.

While oral iron therapy is the first-line treatment for IDA due to its accessibility and low cost, its efficacy is frequently hampered by a variety of variables. Oral iron is not effective in many clinical circumstances due to poor adherence rates, gastrointestinal side effects affecting up to 70% of patients, and insufficient efficacy in moderate-to-severe anemia (14–16). Oral iron absorption is limited, absorbing just 10–20% of consumed iron even under normal conditions (17). This proportion lowers further in the presence of inflammation or concurrent medications (17). International guidelines recommend intravenous iron administration for patients with moderate-to-severe IDA (hemoglobin < 10 g/dL), documented intolerance to oral iron, inadequate hemoglobin increase after adequate oral treatment trial, or clinical situations requiring rapid hemoglobin reconstitution (18–20).

Ferric carboxymaltose (FCM), also known as Ferinject®, is a significant development in intravenous iron therapy. FCM, a third-generation intravenous iron formulation, can deliver up to 1,000 mg of iron in a single 15-min infusion, providing significant practical improvements over prior formulations that needed numerous smaller doses or lengthy infusion times (21, 22). FCM’s iron-carbohydrate complex composition prevents free iron toxicity and promotes efficient iron transport to transferrin and hemoglobin (23). FCM has been shown to be highly effective in a variety of patient populations, including patients with inflammatory bowel disease, chronic renal disease, heart failure, and excessive menstrual bleeding (24–28). The FER-ASAP study found that FCM significantly increased hemoglobin levels compared to oral iron (mean difference 4.2 g/L at 3 weeks, *p* < 0.001). However, the efficacy of FCM therapy is dependent on proper dosing, an important element that has received scant attention in the literature. Calculating the optimal FCM dose is simple when using the manufacturer’s approved dosing recommendations. The Summary of Product Characteristics (SmPC) offers a simple dosing chart based on patient weight and baseline hemoglobin level, reducing the need for complicated calculations (29, 48). Despite clear criteria, anecdotal data and limited research suggest that FCM is commonly administered at inadequate doses, potentially weakening its therapeutic advantages (30).

Insufficient iron replacement has serious clinical effects. Detlefs et al. found that poor iron therapy response led to a higher likelihood of undesirable outcomes, such as prolonged hospitalization and increased healthcare utilization (31). These findings suggest that iron therapy should aim to restore iron deficiency completely using evidence-based dose, not just start treatment.

Two principal strategies exist for estimating the iron requirement in iron deficiency anemia. The classical Ganzoni formula derives the total iron deficit from body weight, the difference between target and actual hemoglobin, and iron stores [total iron deficit (mg) = body weight (kg) × (target Hb − actual Hb; g/dL) × 2.4 + iron stores (mg)] (32). Although precise, this calculation-intensive approach has been associated with systematic under-replacement in routine practice. To simplify dosing, the ferric carboxymaltose Summary of Product Characteristics (SmPC) provides a cumulative-dose table stratified by body weight and baseline hemoglobin, which is now the recommended basis for dosing in most jurisdictions and the reference standard adopted in the present study. The degree to which real-world prescribing adheres to either strategy has rarely been quantified, providing a direct rationale for comparing prescribed against SmPC-calculated cumulative doses.

Beyond efficacy, contemporary attention has increasingly focused on the safety profile of intravenous iron, and of FCM in particular. Although hypersensitivity reactions are uncommon across modern formulations, FCM has been consistently associated with hypophosphatemia, mediated by an FCM-induced rise in intact fibroblast growth factor 23 and consequent renal phosphate wasting. Head-to-head randomized trials have reported hypophosphatemia in a substantially higher proportion of FCM-treated patients than with ferric derisomaltose, and persistent or symptomatic hypophosphatemia, including, in rare cases, osteomalacia and fractures, has been described with repeated exposure (30, 33). These observations underscore that both under-dosing, which compromises efficacy, and the cumulative dose actually delivered, which bears on safety, are clinically consequential and merit real-world characterization.

Despite the extensive usage of ferric carboxymaltose (FCM) in Saudi healthcare institutions, real-world data on prescribing patterns, dosage accuracy, and clinical effects are still limited. This disparity is especially noteworthy given the high prevalence of iron deficiency anemia among Saudi women and potential healthcare-system implications on prescribing habits.

Although randomized trials have established the efficacy of weight- and hemoglobin-based FCM dosing, the extent to which these regimens are reproduced in routine clinical practice remains poorly defined. The few available real-world analyses originate predominantly from European and North American settings and report variable rates of label-discordant dosing (30, 34); comparable data from the Middle East, where iron deficiency anemia is highly prevalent and intravenous iron use is expanding rapidly, are essentially absent. The specific evidence gap addressed here concerns the magnitude, direction, and downstream clinical and economic consequences of FCM dosing deviations in routine care.

The purpose of this study was to assess FCM prescribing at a tertiary general hospital by characterizing patient demographics, assessing dosing appropriateness against manufacturer recommendations, identifying predictors of inappropriate dosing, evaluating hemoglobin response and follow-up timing, investigating the role of pharmacy interventions, and estimating the financial impact of current practices. We hypothesized that, in routine practice, a substantial proportion of FCM prescriptions would deviate from SmPC-recommended cumulative doses; that such deviations would predominantly reflect under-dosing; and that under-dosing would be associated with an attenuated hemoglobin response relative to that reported in adequately dosed populations. Because under-replacement may shift costs from the pharmacy budget to downstream care, we further undertook an exploratory economic analysis to estimate whether apparent medication savings from reduced dispensing are offset by the projected costs of anemia-related complications, thereby informing the value case for dosing optimization.

## 2 Methods

### 2.1 Ethical consideration

The study was approved by the Directorate of Health Affairs’ Institutional Review Board in Jeddah, Saudi Arabia, and is registered with the National Committee of Bioethics at King Abdulaziz City for Science and Technology (NCBE-KACST) under registration number H-02-J-002. The approval was issued by the Local Research Ethics Committee, Ministry of Health, Jeddah (Approval No. A02476), with a waiver of consent granted for this retrospective study.

### 2.2 Study design and setting

This real-world observational study used a retrospective design to capture real-world prescribing practices without influencing prescriber behavior, thereby providing an unbiased assessment of routine clinical practice. The study was carried out at a 300-bed tertiary care general hospital operated by the Saudi Ministry of Health in Saudi Arabia.

### 2.3 Study population and eligibility criteria

The study population comprised all adult patients who received ferric carboxymaltose (Ferinject®) at the study hospital across both inpatient and outpatient settings during a six-month study period beginning on January 20, 2025. This timeframe was selected to ensure an adequate sample size while reflecting contemporary prescribing practices. Inclusion criteria consisted of adult age (≥18 years), receipt of at least one dose of ferric carboxymaltose during the study period, and availability of complete medical records, including documented baseline body weight, baseline hemoglobin level, and at least one follow-up hemoglobin measurement. Patients were excluded if they had incomplete data precluding assessment of dosing appropriateness, missing baseline hemoglobin or weight measurements, or if they were transferred to another healthcare facility before completion of therapy or follow-up assessment.

In this study, the diagnosis of iron deficiency anemia was established by the treating clinical team in accordance with World Health Organization and contemporary guideline criteria. Anemia was defined as a hemoglobin concentration below 13 g/dL in men and below 12 g/dL in non-pregnant women (below 11 g/dL during pregnancy), with iron deficiency supported, where biochemical indices were available, by a serum ferritin < 30 ng/mL (or < 100 ng/mL in the presence of inflammation or chronic disease) and/or transferrin saturation < 20%. Anemia severity was graded using standard WHO thresholds (3, 19). As iron-study panels were not uniformly documented in this retrospective cohort, the diagnosis recorded by the treating team applying these criteria served as the reference for inclusion.

### 2.4 Data collection procedures

Data were retrospectively obtained from the Careware® electronic medical record (EMR) system and pharmacy dispensing information. All patients who received at least one dose during the study period were identified from the hospital pharmacy dispensing records and cross-checked against the electronic medical record. The Careware® system offers extensive clinical documentation and facilitates dependable access to patient and prescriber data for research objectives. The gathered variables encompassed patient demographics (age, body mass index, pregnancy status and trimester), prescriber attributes (department and training level), previous iron therapy, prescribed ferric carboxymaltose (Ferinject®) dosage and frequency, baseline and follow-up hemoglobin levels, timing of hemoglobin reassessment, pharmacy interventions, and the quantity of vials dispensed relative to the expected dosage.

Hemoglobin response was defined as the difference between baseline hemoglobin and the first available follow-up hemoglobin recorded after FCM administration. Because this was a retrospective study, the timing of follow-up testing was determined by routine clinical care rather than by a fixed protocol and therefore varied between patients; the absence of a standardized reassessment interval is acknowledged among the study limitations. Safety information was extracted from the electronic record where documented. However, adverse events were not systematically or prospectively recorded, and routine post-infusion serum-phosphate monitoring was not performed; consequently, the incidences of infusion-related/hypersensitivity reactions and of FCM-associated hypophosphatemia could not be reliably determined.

### 2.5 Appropriateness assessment

Clear operational definitions were established *a priori* to ensure consistency and reproducibility. Appropriateness of prior oral iron therapy was assessed based on dose, frequency, duration, and treatment failure criteria according to international guidelines. Ferric carboxymaltose dosing appropriateness was evaluated by comparing the prescribed cumulative dose (PCD) against the calculated cumulative dose (CCD) derived from the manufacturer’s Summary of Product Characteristics (SmPC) dosing table (Appendix Table 1). Dosing was classified as appropriate when PCD matched CCD, subtherapeutic when PCD was lower than CCD, and supratherapeutic when PCD exceeded CCD. This classification used an exact match rather than a tolerance margin (a prescribed dose equal to the calculated dose was appropriate, lower was subtherapeutic, and higher was supratherapeutic); the complete operational definitions are provided in Appendix 1. Financial impact was assessed from direct medication costs and projected complication costs. Complete definitions and criteria for all appropriateness assessments are provided in Appendix 1. The economic analysis was conducted from the healthcare-payer perspective and was exploratory in nature. Direct medication cost was derived from the institutional acquisition price per 500 mg vial (356.35 SAR). Projected complication costs were modeled for the pregnant subgroup only, by applying published adjusted odds ratios for adverse maternal and neonatal outcomes associated with an inadequate response to iron therapy (31) to assumed baseline event probabilities and multiplying the resulting incremental cases by published per-event treatment costs; currency conversion used a rate of 1 USD ≈ 3.75 SAR. Because complication rates were not directly observed in this cohort, the analysis is presented as a scenario/sensitivity exercise rather than a formal cost-effectiveness model, and the cost inputs, odds-ratio sources, and modeling assumptions are reported alongside the corresponding calculations in the Results.

As an observational, single-center study, the analysis is susceptible to confounding and to biases inherent in retrospective designs. To mitigate confounding in the assessment of predictors of inappropriate dosing, age, body-mass-index category, pregnancy status, care setting, prescriber department, and prescriber level were entered into a multivariable logistic regression model; nonetheless, unmeasured confounders (for example, concurrent blood loss, inflammatory status, and adherence to follow-up) could not be controlled. Selection bias arising from the exclusion of patients with incomplete records was examined by comparing the baseline characteristics of included and excluded patients (Section 3.1). Information bias was minimized through the use of standardized electronic-record and pharmacy-dispensing data sources and the application of *a priori* operational definitions for all appropriateness assessments.

### 2.6 Study objectives

- To describe patient demographic and clinical characteristics.
- To evaluate prescriber characteristics and assess prior oral iron therapy status and appropriateness.
- To assess ferric carboxymaltose dosing appropriateness.
- To determine predictors of inappropriate cumulative dosing.
- To evaluate prescribing patterns by baseline hemoglobin level and care setting.
- To assess hemoglobin response to ferric carboxymaltose therapy.
- To assess dispensing patterns and estimate the financial impact of current prescribing practices.

### 2.7 Statistical analysis

All statistical analyses were carried out using the Statistical Package for Social Sciences (SPSS) version 25.0 (IBM Corporation, Armonk, NY, USA). The Shapiro–Wilk test and a visual examination of histograms were used to determine normality in continuous variables. Normally distributed continuous variables were presented as means ± standard deviation (SD), while non-normally distributed variables were reported as medians with interquartile ranges (IQR). Categorical variables are represented as frequencies and percentages.

To compare PCD and CCD, independent samples t-tests were used. Paired samples t-tests were used to compare hemoglobin levels before and after therapy within patients. For categorical variables, Fisher’s exact test was used due to the small sample size in some cells, with the chi-square test applied when all expected cell counts exceeded 5. Binary logistic regression was employed to identify independent predictors of inappropriate cumulative dosing. Results were reported as odds ratios (OR) with 95% confidence intervals (CI). A two-tailed *p*-value of less than 0.05 was regarded as statistically significant for all analyses. The multivariable logistic-regression model included 6 covariates with 14 events in the limiting (appropriately dosed) group, yielding an events-per-variable ratio of approximately 2.3 well below the widely recommended minimum of 10. The model is therefore regarded as hypothesis-generating only, and all odds ratios are reported solely to indicate the direction of effect. No formal *a priori* sample-size calculation was performed; all eligible patients during the study window were included, and the achieved sample size (*n* = 79) was therefore one of convenience. Given the limited number of inappropriately dosed events relative to the number of candidate predictors, the multivariable logistic regression was regarded as exploratory; to limit overfitting, the number of covariates was constrained, and odds ratios are reported with 95% confidence intervals and interpreted with emphasis on the precision of the estimate rather than on statistical significance alone. Missing data were handled by complete-case analysis, with patients lacking baseline or follow-up data excluded rather than imputed (Section 3.1 and Limitations). For key continuous comparisons, mean differences with 95% confidence intervals are reported alongside *p*-values so that the magnitude of effects, and not solely their statistical significance, can be appraised.

## 3 Results

### 3.1 Patient selection and demographics

**Figure 1:**
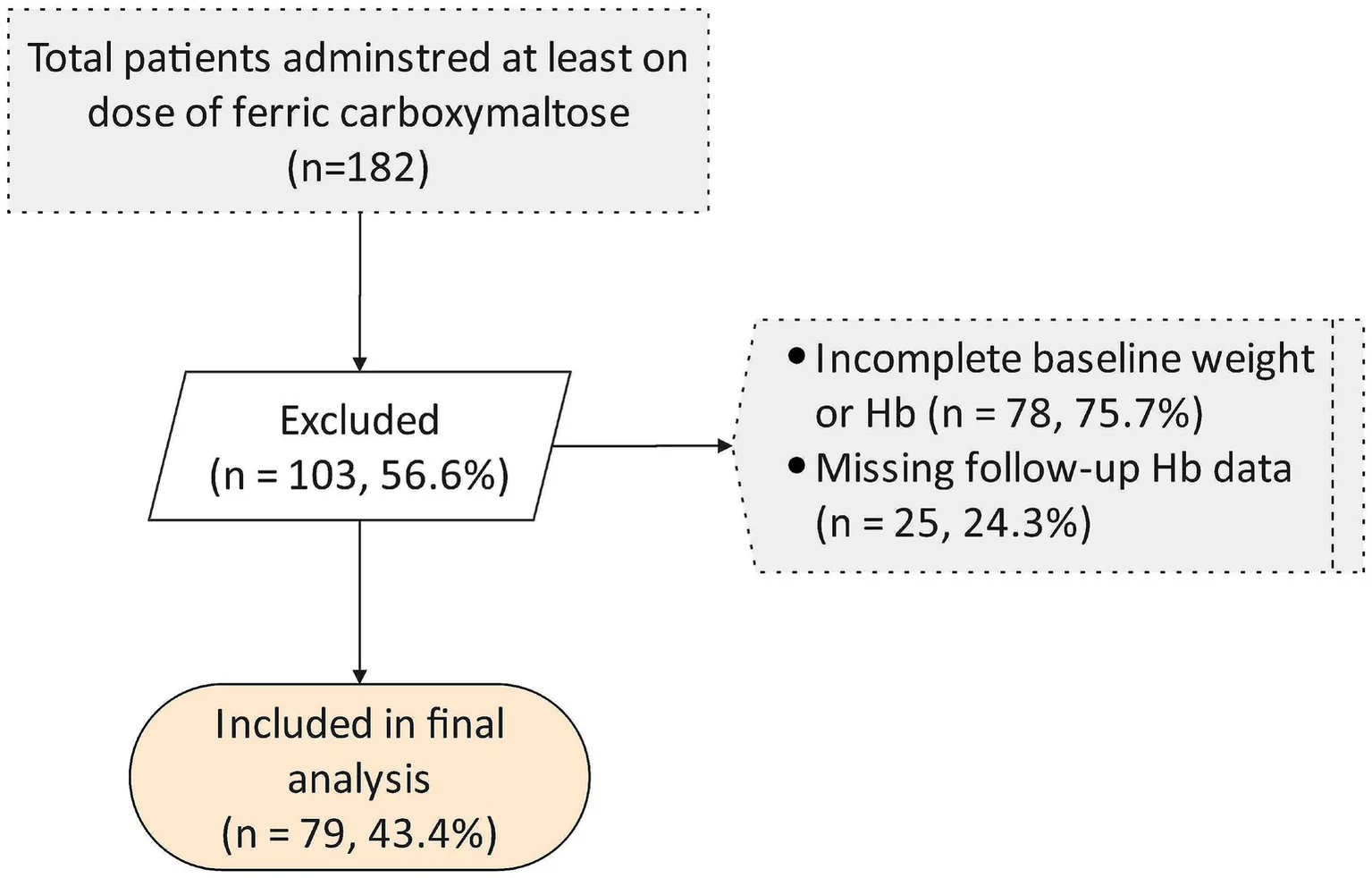
Flow diagram for the inclusion of study patients. FCM, ferric carboxymaltose; Hb, hemoglobin.

Over the six-month study duration, 182 patients were administered at least one dose of ferric carboxymaltose at the study hospital. Following the application of inclusion and exclusion criteria, 103 patients (56.6%) were excluded: 78 (75.7%) for incomplete documentation of baseline weight or hemoglobin, and 25 (24.3%) for absent follow-up hemoglobin data. The total analytical cohort consisted of 79 patients (Figure 1). As a sensitivity exercise, we considered the most conservative scenario in which all 103 excluded patients had been appropriately dosed (0% inappropriate). Even under this assumption, the overall rate of inappropriate dosing across all 182 patients would be 82% × 79/182 = 35.6%, which remains clinically substantial; conversely, if the excluded patients had shared the same 82% rate, the overall estimate would be unchanged. The direction and clinical significance of the main finding are therefore robust to the exclusion pattern.

The demographic and clinical characteristics of the study population are summarized in Supplementary Table 1. The cohort was predominantly female (97.5%, n = 77). The mean age was 34.60 ± 8.32 years (range: 19–52 years). The mean BMI was 27.90 ± 6.05 kg/m^2^. Based on BMI classification, 2.5% of patients were underweight (BMI < 18.5 kg/m^2^), 29.1% had normal weight (BMI 18.5–24.9 kg/m^2^), 35.5% were overweight (BMI 25.0–29.9 kg/m^2^), and 25.3% were obese (BMI ≥ 30 kg/m^2^). BMI could not be calculated for the remaining 6 patients (7.6%) owing to incomplete height data. Pregnant women constituted 34.2% (*n* = 27) of the study population. Among pregnant patients, 1.3% were in the first trimester (*n* = 1), 5.1% in the second trimester (*n* = 4), and 27.8% in the third trimester (*n* = 22). Iron deficiency anemia was confirmed as the primary indication in 94.9% of cases.

Regarding care setting, the majority of FCM prescriptions were initiated in the outpatient setting (72.2%, *n* = 57), compared to inpatient setting (27.8%, *n* = 22). The mean baseline hemoglobin was 8.99 ± 1.54 g/dL, classifying the majority of patients as having moderate anemia (hemoglobin 7.0–9.9 g/dL) according to WHO criteria. The mean total cumulative dose administered was 1,118 ± 492 mg.

### 3.2 Prescriber characteristics and previous Iron therapy

The distribution of prescribers by department indicated that the predominant source of FCM prescriptions was Obstetrics and Gynecology (74.7%), followed by the Emergency Department (12.7%) and Internal Medicine (8.9%), and other departments (3.7%). Residents constituted the predominant share of prescriptions at 54.4%, followed by consultants at 30.4% and specialists at 15.2%.

**Figure 2:**
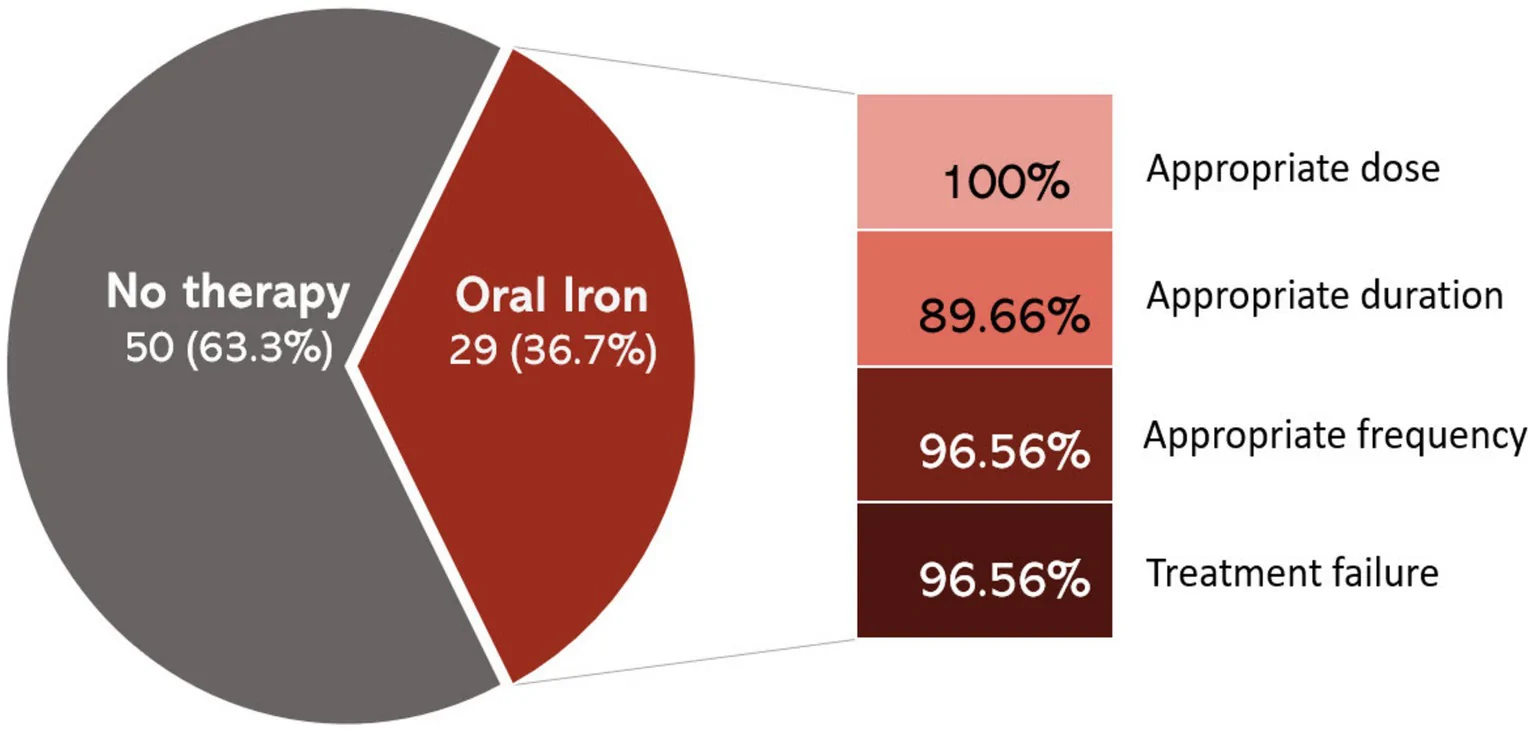
Prior oral iron therapy and appropriateness assessment (*N* = 79).

**Figure 3:**
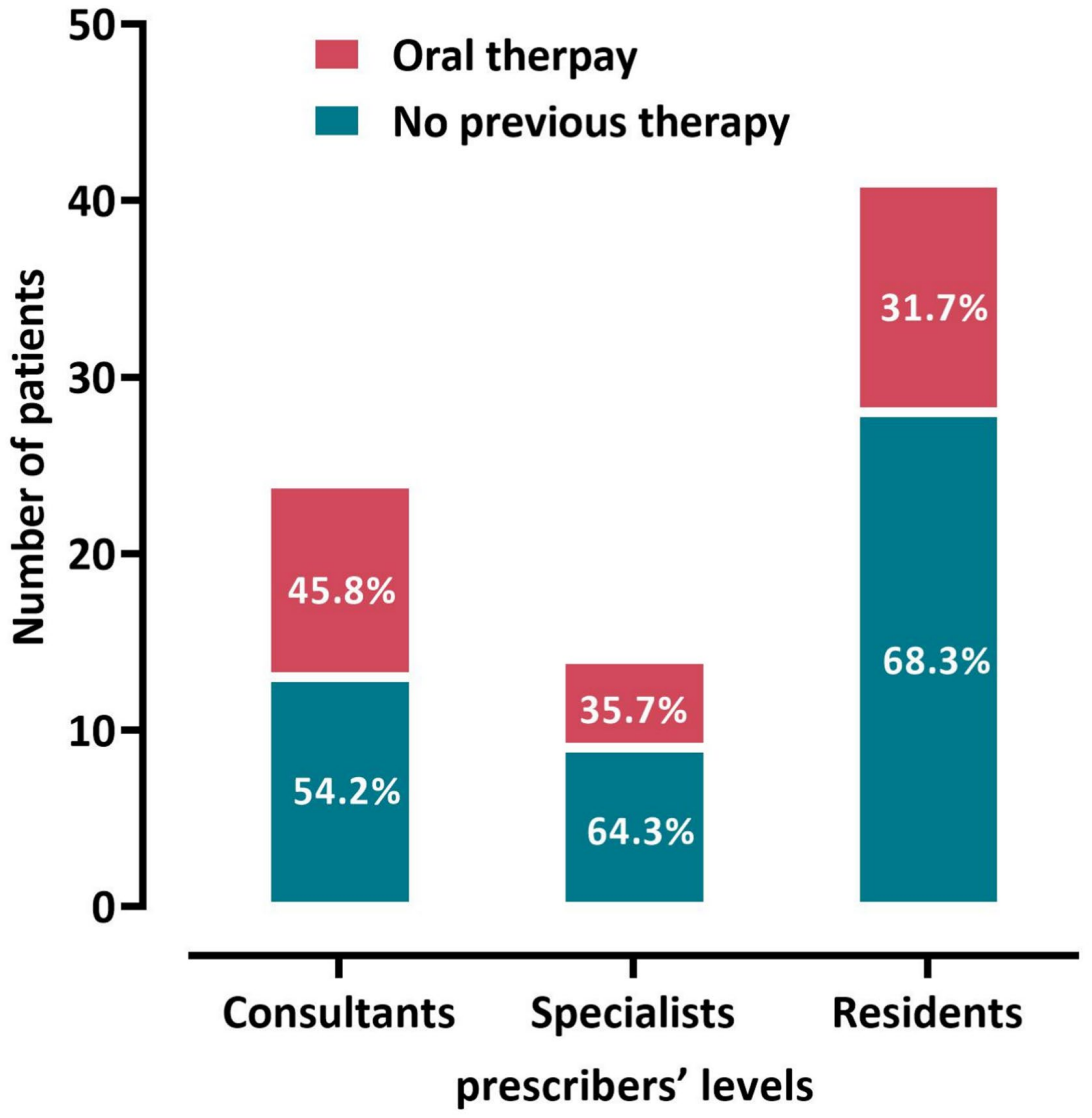
Prior oral iron therapy by prescriber level (*N* = 79).

Analysis of prior iron therapy indicated that 63.3% (*n* = 50) of patients were naïve to iron therapy at the start of FCM administration. Of the 36.7% (*n* = 29) who received prior oral iron therapy, evaluation of the oral iron regimen according to our established criteria revealed that 100% received suitable dosages, 89.66% adhered to the acceptable duration, and 96.56% followed the appropriate frequency. In 96.56% of these patients, treatment failure occurred despite the administration of proper oral iron therapy (Figure 2). Logistic regression analysis demonstrated no significant association between prescriber level and the likelihood of prescribing ferric carboxymaltose to patients without prior iron therapy (Omnibus χ^2^ = 1.293, df = 2, *p* = 0.524). When using consultants as the reference group, specialists showed a non-significant elevation in the likelihood of prescribing ferric carboxymaltose to iron-therapy-naïve patients (OR = 1.52, *p* = 0.543), but residents also displayed increased odds relative to consultants (OR for iron-naïve prescribing = 1.82, *p* = 0.257). Nonetheless, neither comparison attained statistical significance (Figure 3).

Stacked bar chart showing the proportion of patients with prior oral iron therapy (pink) vs. no previous therapy (teal) by prescriber level. Residents had the highest rate of prescribing FCM to iron-therapy naïve patients (68.3%), compared to specialists (64.3%) and consultants (54.2%).

### 3.3 Dosing appropriateness assessment

Among the 79 analyzed FCM prescriptions, the majority (86%) were deemed inappropriate regarding duration and/or frequency. In individual dose evaluation, almost one-third (33%) of first doses were deemed incorrect; however, a significantly greater proportion (83%) of subsequent doses were inappropriate. In total, 82% of prescriptions exhibited improper cumulative dosages when assessed against the calculated cumulative dose (CCD). Of the inappropriately dosed prescriptions, a significant majority (88%) were subtherapeutic, whereas only 12% were supratherapeutic.

**Figure 4:**
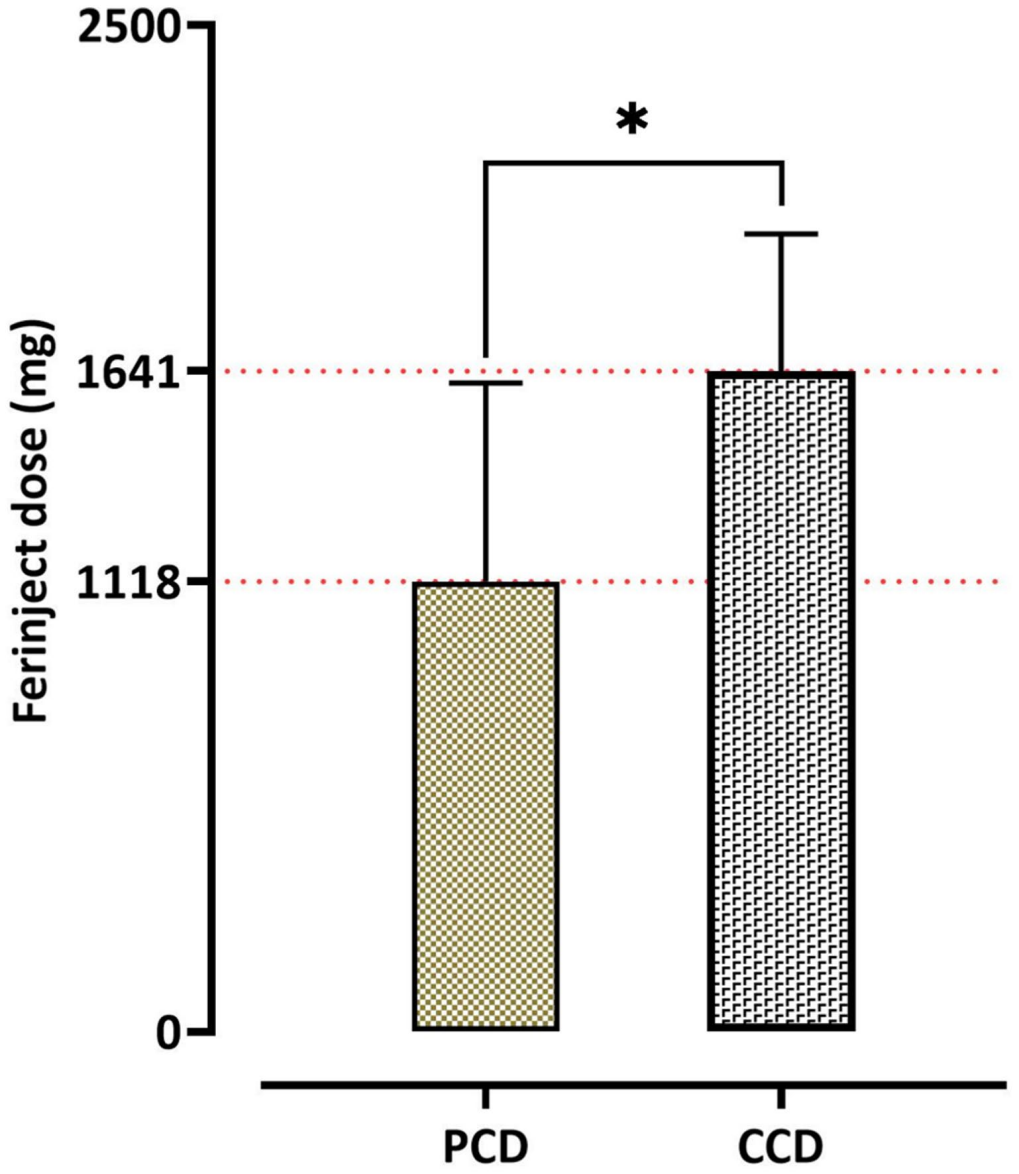
Comparison between prescribed cumulative dose (PCD) and calculated cumulative dose (CCD) of ferric carboxymaltose. The bar chart illustrates the mean prescribed cumulative dose (PCD) and the calculated cumulative dose (CCD) of ferric carboxymaltose among the study population. Error bars represent standard deviation. Values are expressed as means ± SD. PCD represents the prescribed cumulative dose, and CCD represents the calculated cumulative dose. *p < 0.001 between PCD and CCD. Statistical analysis was performed using an independent t-test.

Quantitative comparison between prescribed cumulative dose (PCD) and calculated cumulative dose (CCD) revealed a highly significant difference (*p* < 0.001). The mean PCD was 1,118 ± 492 mg compared to mean CCD of 1,640 ± 438 mg, representing a mean under-dosing gap of 522.6 ± 69.86 mg (95% CI: 506.8–538.4 mg; Figure 4).

### 3.4 Predictors of inappropriate dosing

**Figure 5:**
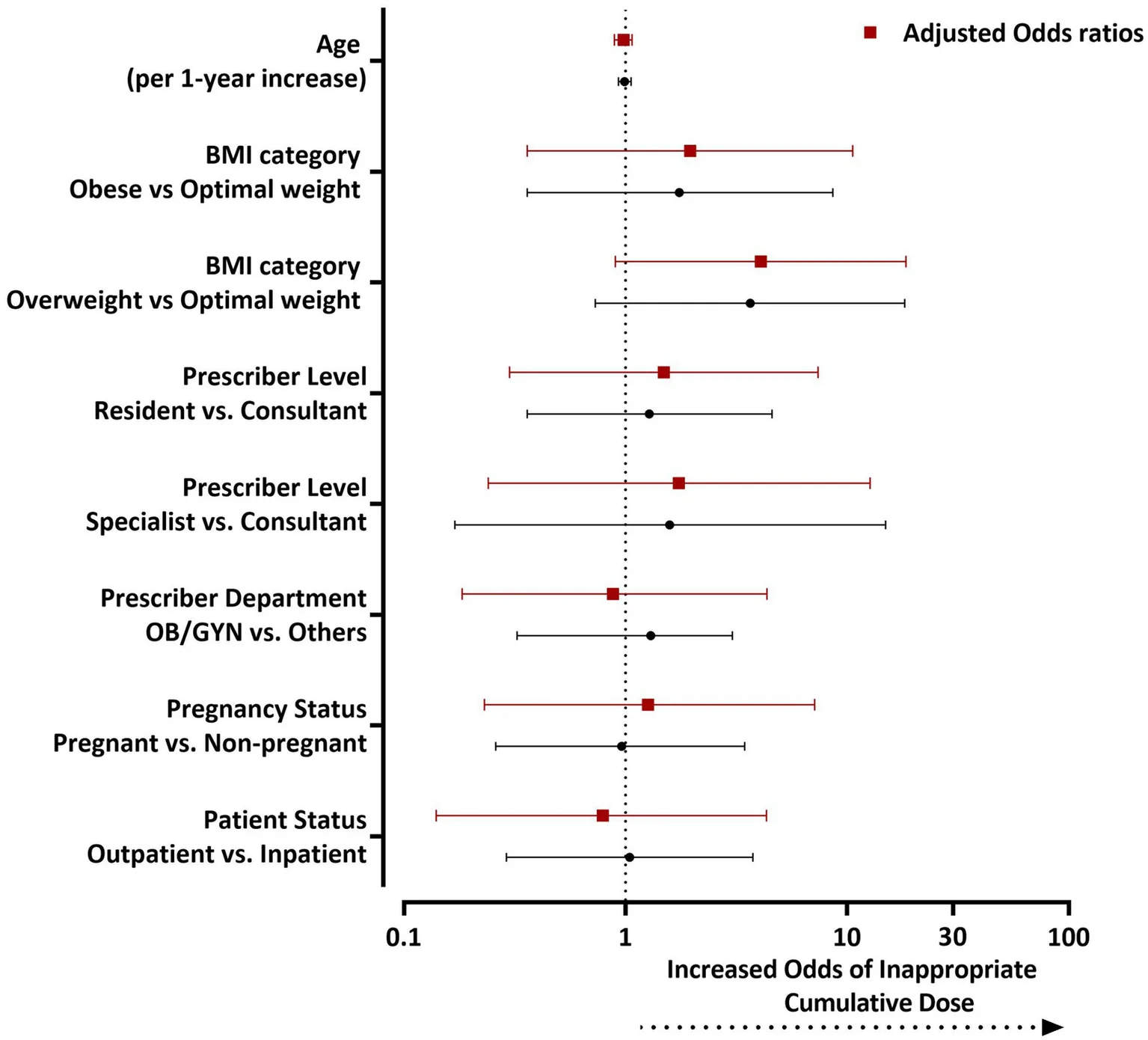
Forest plot of factors associated with inappropriate cumulative ferric carboxymaltose dosing. The forest plot displays unadjusted (black circles) and adjusted (red squares) odds ratios with corresponding 95% confidence intervals for demographic, clinical, and prescribing-related factors associated with inappropriate cumulative ferric carboxymaltose dosing. The vertical dotted line represents the null value (odds ratio = 1). Odds ratios to the right of the reference line indicate increased odds of inappropriate dosing. All adjusted estimates were derived from the multivariable logistic regression model.

Univariable and multivariable logistic regression analyses were conducted to identify characteristics linked to inappropriate cumulative dosing of ferric carboxymaltose (Figure 5). In unadjusted analyses, no demographic, clinical, or prescribing-related variables exhibited a significant association with dosage appropriateness. Following adjustment, patient status, pregnancy status, prescriber department, prescriber level, BMI category, and age were found to be non-significant predictors. While overweight individuals exhibited greater adjusted odds of incorrect dose relative to those of ideal weight (adjusted OR 4.08, 95% CI 0.90–18.44), this association did not achieve statistical significance. Other adjusted estimates were likewise non-significant, including residents vs. consultants (adjusted OR 1.49, 95% CI 0.30–7.40) and the outpatient vs. inpatient setting (adjusted OR 0.79, 95% CI 0.14–4.32); all adjusted estimates are presented in Figure 5.

### 3.5 Prescribing patterns by hemoglobin level and setting

Among outpatients, 16% of ferric carboxymaltose prescriptions were for patients with hemoglobin levels ≥10.5 g/dL, compared with only 4.5% of inpatient prescriptions. Fisher’s exact test demonstrated that outpatients were 3.8 times more likely to receive FCM with hemoglobin ≥10.5 g/dL compared to inpatients (OR 3.8, 95% CI: 0.62–44.18, *p* = 0.16).

### 3.6 Hemoglobin response

Paired-samples analysis demonstrated a significant increase in hemoglobin levels following ferric carboxymaltose administration. The mean hemoglobin increased from 8.84 ± 1.50 g/dL at baseline to 10.20 ± 1.79 g/dL at follow-up (mean difference = +1.35 g/dL; 95% CI: 0.81–1.90, *p* < 0.001).

### 3.7 Dispensing patterns and financial impact

Based on the CCD calculations, the expected total consumption was 149 vials; however, only 94 vials were actually dispensed. This resulted in a shortfall of 55 vials, corresponding to a 36.9% under-dispensing rate. This apparent under-dispensing translated into a false cost saving of 19,599.25 SAR (55 vials × 356.35 SAR per vial).

Among pregnant women, 85% (17 out of 20) received subtherapeutic doses. Using published odds ratios for inadequate iron therapy response, projected complication rates were calculated. For preeclampsia, the adjusted probability increased from 5% baseline to 7.2%, yielding 1.22 expected cases (7.2% × 17). The estimated treatment cost for preeclampsia was $11,454.58 (≈ 42,955 SAR; 1.22 cases × $9,389). For preterm birth, the adjusted probability increased from 10% baseline to 14.4%, yielding 2.44 expected cases (14.4% × 17). The estimated treatment cost for preterm births was $34,160.00 (≈ 128,100 SAR; 2.44 cases × $14,000 per case), resulting in a total projected financial loss of $45,614.58 (≈ 171,055 SAR).

## 4 Discussion

This is the first comprehensive study of ferric carboxymaltose prescribing patterns in a Saudi healthcare facility. Obstetrics & Gynecology prescribed the most FCM (74.7%), consistent with Saudi Arabia’s high iron deficiency anemia rate in reproductive-age women (9, 35). Residents (54.4%) issued the most prescriptions, followed by consultants (30.4%) and specialists (15.2%). According to the conventional hierarchy of prescribing at tertiary care centers, residents under supervision write the most frequent prescriptions (36). Similar patterns have been found in other healthcare settings. Although the EQUIP study examined general prescribing errors by foundation trainees rather than FCM-specific dosing, its reported junior-doctor error rate of 8.4–10.3% (vs. 5.9% for consultants) provides contextual background (37). The FCM-specific analyses by Schaefer et al. and Aksan et al. remain the more directly relevant comparators for the dosing deviations observed in this study. The high rate of incorrect doses may be due to resident prescribers’ lack of knowledge of speciality medications and product-specific dosing needs. Velo and Minuz found that junior workers make more mistakes, and that low understanding or training causes inappropriate prescribing (38). Systematic reviews have indicated that focused educational programs can improve junior doctors’ prescription competency; therefore, targeting this group could improve prescribing quality (39).

A significant finding was that 63.3% of patients were iron-therapy naïve at the time of FCM initiation. This raises serious concerns about adherence to treatment standards, as international recommendations consistently call for a trial of oral iron therapy before escalating on to intravenous formulations in most clinical situations. The British Society of Hematology recommends prescribing oral iron as first-line therapy, with intravenous iron reserved for individuals with demonstrated intolerance, noncompliance, or unsatisfactory response after 4 weeks of suitable oral medication (40). Similarly, the American College of Obstetricians and Gynecologists recommends oral iron supplementation as the first treatment for iron deficiency anemia in pregnancy, with parenteral iron recommended when oral therapy is inadequate or not tolerated (41). While there may be proper clinical reasons for bypassing oral iron in certain cases such as moderate-to-severe anemia requiring rapid correction, anticipated poor absorption due to gastrointestinal conditions or logistical challenges with oral therapy adherence, the high proportion of treatment-naïve patients in our study suggests that oral iron may have been underutilized. This pattern of premature escalation to intravenous therapy raises healthcare expenses while providing no demonstrable benefit over proper oral iron therapy in uncomplicated patients.

Among patients who had previously received oral iron therapy, our assessment using predefined criteria revealed reassuringly high rates of adequate dose (100%), duration (89.66%), and frequency (96.56%). Treatment failure despite proper oral iron therapy was documented in 96.56% of these patients, demonstrating that when oral iron was prescribed, it was administered correctly, and escalation to FCM was justified. This finding indicates that the problem is not incorrect oral iron prescribing, but rather a tendency to skip this step entirely. A logistic regression analysis found no significant relationship between prescriber level and the chance of prescribing FCM to iron-therapy naïve individuals (*p* = 0.524), indicating that this pattern occurred across all prescriber levels rather than being driven by a single group. The comparable odds ratios for specialists (OR 1.52) and residents (OR 1.82) compared to consultants indicate that systemic issues, rather than individual prescriber characteristics, may be driving this behavior.

The study’s main and most troubling conclusion was that 82% of FCM prescriptions contained improper cumulative doses when compared to the manufacturer’s SmPC dosing table. The great majority of incorrectly dosed prescriptions (88%) were subtherapeutic, with only 12% being supratherapeutic. The prescribed cumulative dose of 1,118 ± 492 mg was substantially lower than the calculated cumulative dose of 1,640 ± 438 mg, indicating a mean under-dosing gap of 522.6 mg (*p* < 0.001). This discrepancy is clinically significant, as it represents about one more 500 mg vial that should have been provided to obtain the recommended cumulative dose. Based on clinical trial data, a 500 mg dosage of FCM is projected to improve hemoglobin by about 1.0–1.5 g/dL in an iron-deficient patient (42), suggesting that the observed under-dosing likely compromised hemoglobin recovery by a similar magnitude.

Comparing our findings to published literature demonstrates that incorrect FCM dose is not unique to our institution. A European study by Schaefer et al. indicated that around 30% of FCM prescriptions in primary care were inconsistent with the labeling instructions (30). A United States study examining FCM prescribing patterns in inflammatory bowel disease found that 33% of patients received less than 1,000 mg total iron, and this discordance was associated with increased healthcare utilization (34). The significantly greater prevalence of inappropriate dosing found in our study (82% vs. earlier reports) could be attributed to variations in healthcare system structure, availability of clinical decision support systems, prescriber training, or patient population characteristics. Notably, the consistent finding across several studies that under-dosing is significantly more common than over-dosing shows a systemic bias toward conservative prescribing, which, while well-intended, ultimately compromises optimal treatment outcomes.

Numerous variables may underlie widespread under-dosing. First, prescriber unfamiliarity with the SmPC dose table may be important. Even though the manufacturer provides a weight-based and hemoglobin-based dose table that removes difficult calculations, prescribers may not always examine product information. Second, the prevalence of under-dosing shows prescribers may choose lower doses for safety or financial reasons. This strategy ignores practical hurdles to follow-up, such as patient non-return for further doses and clinical worsening between inadequate treatments. Third, the electronic prescribing system lacks integrated clinical decision support; thus, prescribers receive no real-time dose input. A simple electronic alert showing the cumulative dose based on patient weight and hemoglobin could greatly improve dosing accuracy.

The SmPC cumulative-dose table was used as the reference standard because it is the label-recommended method and a simplified, banded approximation of the Ganzoni iron deficit; the two give broadly comparable targets, the table often slightly higher at greater body weights. Because both scale dose with body weight and the calculated cumulative dose used each patient’s actual weight, the higher targets for heavier patients were fully captured, which may explain the higher (non-significant) odds of inappropriate dosing in overweight patients. A Ganzoni-based re-analysis would likely narrow the under-dosing gap only modestly without reversing its direction, as prescribed doses fell well below either target in most cases.

A single ferric carboxymaltose administration is capped at 1,000 mg (and 20 mg/kg) per week, so any deficit above 1,000 mg requires more than one visit, unlike formulations such as iron polymaltose that can deliver a larger deficit in one infusion. Much of the observed under-dosing therefore likely reflects an appropriate first dose followed by non-completion of the remaining course rather than miscalculation, consistent with the first-dose (33%) vs. subsequent-dose (83%) error differential. The proportion given only the maximum single dose vs. those whose total deficit was itself below 1,000 mg could not be separated from the available records and is a priority for prospective study.

This interpretation is reinforced by the contrast between the first-dose error rate (33%) and the subsequent-dose error rate (83%). The lower first-dose error rate suggests that initial dose selection informed by the SmPC table at the point of prescribing was more often correct, whereas the markedly higher subsequent-dose error rate points predominantly to incomplete course completion rather than recurrent calculation failure. The two error patterns therefore call for distinct remedies: the decision-support alert described above to address first-dose miscalculation, and, in addition, a structured patient-recall or electronic-reminder system to prevent premature discontinuation of the planned treatment course.

Predictors of incorrect cumulative dose were identified using multivariable logistic regression. None of the demographic, clinical, and prescriber factors were statistically significant in the adjusted model. Overweight patients had greater adjusted odds of incorrect dose than ideal weight individuals (adjusted OR 4.08, 95% CI: 0.90–18.44, *p* = 0.090), although this association was not statistically significant. Residents had higher odds than consultants (adjusted OR for inappropriate dosing 1.49, 95% CI: 0.30–7.40, *p* = 0.625) and outpatients had lower odds than inpatients (adjusted OR 0.79, 95% CI: 0.14–4.32, *p* = 0.788), but neither approached significance. There were no significant predictors, suggesting that incorrect dose was widespread across patient and prescriber groupings, indicating a systemic issue. Quality improvement efforts should target the prescribing system as a whole through clinical decision support, standardized protocols, and universal prescriber education, not just high-risk groups.

A surprising finding was that 16% of FCM prescriptions were for patients with hemoglobin levels ≥10.5 g/dL, raising concerns regarding the suitability of intravenous iron therapy for moderate anemia. International guidelines generally recommend intravenous iron for patients with moderate-to-severe anemia (hemoglobin < 10 g/dL) who have failed oral iron therapy (18–20). Clinical decision-making processes may differ between treatment settings. Although the point estimate suggested that outpatients were more likely to undergo FCM with hemoglobin ≥10.5 g/dL than inpatients, this association was not statistically significant and was estimated with very low precision (OR 3.8, 95% CI: 0.62–44.18, *p* = 0.16), and is therefore reported with caution. Inpatient prescriptions may be based on acute clinical need and lower hemoglobin thresholds, while outpatient prescriptions may be based on patient convenience, symptom severity, or iron deficiency without anemia that requires treatment. Intravenous iron is controversial in iron deficiency without anemia; however, it may help symptomatic individuals with heart failure or persistent fatigue (43). Nevertheless, the relatively high proportion of patients with mild anemia receiving FCM warrants further evaluation to ensure appropriate patient selection. This may reflect symptom-driven prescribing, treatment of iron deficiency without anemia, preference for a rapid single-visit therapy, manufacturer promotion of intravenous iron, or the absence of local, operationalized hemoglobin thresholds for intravenous iron use; disentangling these is a priority for local audit.

Although subtherapeutic dosing was common, paired-sample analysis showed a significant increase in hemoglobin levels after FCM administration, with a mean increase of 1.35 g/dL (95% CI: 0.81–1.90, *p* < 0.001). While statistically significant, this response is far lower than that in clinical trials with proper dosing. The FER-ASAP trial showed 2.5–3.0 g/dL hemoglobin gains at protocol-specified dosages of FCM after 3–4 weeks (42). A direct within-cohort comparison of hemoglobin response between appropriately and sub-therapeutically dosed patients could not be robustly performed, because the predominance of subtherapeutic dosing left too few appropriately dosed patients for a stable subgroup estimate; this comparison is proposed as a priority for an adequately powered prospective study. Similarly, real-world studies from European centers with appropriate dosing protocols reported mean hemoglobin increases of 2.5–3.0 g/dL (44–46). The attenuated response in our cohort (1.35 g/dL vs. 2.5–3.0 g/dL in appropriately dosed populations) likely reflects the inadequate dosing documented in this study, providing real-world evidence that under-dosing relative to SmPC recommendations translates directly to inferior clinical outcomes. The observed mean hemoglobin rise of 1.35 g/dL is lower than the 2.5–3.0 g/dL reported in protocol-defined trials with adequate dosing; however, given the variable follow-up timing and population differences in this retrospective cohort, this comparison is descriptive and hypothesis-generating rather than causally attributive.

Analysis of dispensing patterns showed a large discrepancy between calculations and practice. Based on calculated cumulative dose, 149 vials were expected but only 94 were dispensed, a 36.9% shortage. Under-dispensing resulted in cost savings of 19,599.25 SAR (55 vials x 356.35 SAR/vial). This is a false economy that ignores the downstream costs of poor treatment.

Financial research shows the full cost of underdosing. Reduced medication dispensing appears to save money, but it actually costs more due to additional healthcare visits for anemia symptoms, repeated laboratory monitoring, retreatment when anemia returns or fails to resolve, additional diagnostic workup for persistent symptoms, and blood transfusions in severe cases. We anticipated large complication costs for the pregnant subgroup with published outcome data using odds ratios from Detlefs et al. for poor iron therapy response (31). Adjusted probabilities yielded 1.22 expected preeclampsia cases (treatment cost $11,454.58) and 2.44 expected preterm births (treatment cost $34,160.00) in 17 under-dosed pregnant women (85% of 20 pregnant patients), resulting in a projected financial loss of $45,614.58 (approximately 171,055 SAR). This anticipated complication cost surpasses drug savings by a factor of 8.7, resulting in a large financial loss. Unfortunately, there is no available outcome-specific cost data for non-pregnant patients; thus, these calculations only include 34% of patients. This suggests that the true financial burden is significantly underestimated.

FCM is an expensive medicine, and resource-constrained healthcare organizations should consider cost when formulary decisions are made. However, FCM cost-effectiveness depends only on SmPC table dosing. A properly dosed course that corrects anemia and restores functional ability is good value; an insufficiently dosed course that fails to correct anemia and incurs medication costs is poor value. Our data show that current prescribing practices may result in the worst economic outcome: spending money on FCM without realizing its therapeutic benefits. According to a US study, anemic patients had 54% higher overall healthcare costs ($14,535 vs. $9,451) than non-anemic matched controls (47). Ferric carboxymaltose also costs substantially more per milligram of iron than older formulations such as iron polymaltose or iron sucrose, its advantage being rapid single-visit delivery; when it is under-dosed that advantage is lost while the higher cost is still incurred, so where speed is not required a cheaper formulation dosed to the full deficit may offer better value.

Several limitations should be acknowledged. First, the retrospective design introduces inherent limitations including potential selection bias from incomplete records, inability to capture all relevant clinical variables, and reliance on documented rather than actual practice. The exclusion of 56.6% of patients due to incomplete data may have introduced bias if excluded patients differed systematically in their dosing patterns. Second, the single-center design limits generalizability to other Saudi healthcare facilities with different protocols, training programs, or patient populations. Third, the relatively small sample size (*n* = 79) limited statistical power and may explain why no significant predictors of inappropriate dosing were identified despite clinically meaningful differences in odds ratios. Fourth, we could not assess long-term outcomes such as anemia recurrence or retreatment need, which would provide definitive evidence of clinical impact. Fifth, projected complication costs were calculated only for the pregnant subgroup, likely underestimating true financial impact. Sixth, pharmacy intervention data were limited, precluding detailed analysis of medication safety system performance. Seventh, given the small number of inappropriately dosed events relative to the candidate predictors, the multivariable model is statistically underpowered; the odds ratios reported (including the elevated point estimates for overweight status and for the outpatient setting) are imprecise, have wide confidence intervals that cross unity, and should be interpreted as hypothesis-generating rather than as evidence of true associations. Eighth, the economic estimates are model-based and exploratory rather than derived from observed complications; because they depend on a small pregnant subgroup (*n* = 17 under-dosed) and on transferred external probabilities and costs, they are sensitive to the underlying assumptions and are intended to illustrate potential magnitude only. In addition, because the analysis was retrospective, the diagnosis of iron deficiency relied on the documented assessment of the treating team without uniform availability of confirmatory iron studies, and the timing of follow-up hemoglobin measurement was determined by routine clinical care rather than by a fixed protocol, introducing heterogeneity into the response assessment. Finally, safety outcomes, in particular serum-phosphate monitoring for FCM-associated hypophosphatemia, were not systematically recorded, precluding a robust safety appraisal; prospective evaluation incorporating standardized phosphate monitoring is warranted.

## 5 Conclusion

This study identifies major deficiencies in the prescribing of ferric carboxymaltose in a Saudi tertiary hospital, with 82% of prescriptions exhibiting inappropriate cumulative dosing—mainly subtherapeutic—and 63.3% of patients skipping the prescribed oral iron trial. The mean under-dosing gap of 522.6 mg resulted in a diminished hemoglobin response (1.35 g/dL vs. to 2.5–3.0 g/dL in adequately dosed individuals). This comparison is descriptive and hypothesis-generating rather than causally attributive. Inappropriate dosing was prevalent across the cohort; however, no predictor reached statistical significance and, given the low events-per-variable ratio (≈2.3), this should be interpreted as indeterminate rather than as evidence of uniformly distributed risk across prescriber levels or patient groups. The findings have substantial implications: the savings from under-dispensing (19,599 SAR) are negligible compared to the anticipated expenses of complications (171,055 SAR), especially in pregnant women. The single-center design and inadequate records restrict generalizability. The immediate implementation of computerized clinical decision support that incorporates SmPC dose tables, along with focused prescriber education, is essential to optimize FCM utilization and guarantee that patients receive evidence-based iron repletion.

## Data Availability

The data analyzed in this study will be made available by the authors upon reasonable request. Requests to access these datasets should be directed to Yahya A. Alzahrani, yaaalzahrani1@kau.edu.sa.
