# Supplementary material for: Prescribing and dosing patterns of ferric carboxymaltose for iron deficiency anemia: a real-world study from Saudi Arabia
Source: Front Med (Lausanne). 2026 Aug 7;13:1864516. doi: 10.3389/fmed.2026.1864516 (PMC13493227; doi:10.3389/fmed.2026.1864516)
Supplement: Supplementary file 1 [file Table_1.DOCX]

**Appendix 1: Definitions and Criteria for Appropriateness Assessment**

Clear operational definitions were established a priori for all appropriateness assessments to ensure consistency and reproducibility.

**Assessment of Previous Oral Iron Therapy:**

- Appropriate Oral Iron Dose: Oral iron therapy was deemed correctly dosed if the patient obtained elemental iron within the advised therapeutic range. The recommended dosage of ferrous sulphate is 60-200 mg of elemental iron per day, corresponding to 300-1000 mg of ferrous sulphate. The recommended dosage of ferrous fumarate is 60-200 mg of elemental iron daily, corresponding to 180-600 mg of ferrous fumarate. The recommended dosage for ferrous gluconate is 60-200 mg of elemental iron daily, corresponding to 500-1600 mg of ferrous gluconate. These limits align with international guidelines advocating for 100-200 mg of elemental iron daily for the treatment of iron deficient anaemia [1]
- Appropriate Oral Iron Frequency: Oral iron frequency was considered appropriate if administered once daily, twice daily, or three times daily, consistent with standard prescribing recommendations. Alternate-day dosing was also considered appropriate based on emerging evidence of improved absorption with this regimen[1].
- Appropriate Oral Iron Duration: Oral iron therapy duration was considered appropriate if the patient received a minimum of 4 weeks of therapy before being classified as a treatment failure. This timeframe allows sufficient time to assess hematological response, as hemoglobin typically increases by 1-2 g/dL within 2-4 weeks of initiating appropriate oral iron therapy [2].
- Oral Iron Treatment Failure: Treatment failure was defined as: (1) failure to achieve a hemoglobin increase of ≥1 g/dL after 4 weeks of appropriate oral iron therapy (non-response); (2) documented intolerance due to gastrointestinal side effects leading to discontinuation or inability to maintain appropriate dosing (intolerance); or (3) documented non-adherence to prescribed oral iron regimen confirmed by patient interview or pill count (non-adherence).

Assessment of Ferric Carboxymaltose Dosing:

- Calculated Cumulative Dose (CCD): The appropriate total iron requirement was determined using the simplified dosing table from the ferric carboxymaltose Summary of Product Characteristics (SmPC), which is the manufacturer-approved dosing recommendation[3]. This weight-based and hemoglobin-based approach determines the total iron need as shown in appendix table1.
- Appropriate FCM Single Dose: A single FCM administration was considered appropriate if it did not exceed: (1) 15 mg iron per kg body weight for intravenous injection administration; (2) 20 mg iron per kg body weight for intravenous infusion administration; and (3) an absolute maximum of 1,000 mg iron (equivalent to 20 mL Ferinject or two 500 mg vials) per administration[3].
- Appropriate FCM Dosing Frequency: FCM dosing frequency was considered appropriate if a minimum interval of 7 days was maintained between consecutive doses, as specified in the SmPC. The maximum cumulative dose per week should not exceed 1,000 mg iron[3].
- Appropriate FCM Duration/Course Completion: FCM therapy duration was considered appropriate if the patient received the complete calculated cumulative dose. For patients requiring more than 1,000 mg total iron, this necessitated at least two administrations separated by a minimum of 7 days[3].
- Prescribed Cumulative Dose (PCD): The total amount of iron (in mg) actually prescribed to the patient across all FCM administrations.

Dosing appropriateness was classified by comparing the prescribed cumulative dose (PCD) of ferric carboxymaltose with the calculated cumulative dose (CCD) based on the Summary of Product Characteristics (SmPC) dosing table. Dosing was considered appropriate when the PCD exactly matched the CCD. Subtherapeutic dosing (under-dosing) was defined as a PCD lower than the CCD, indicating that the patient received less iron than recommended. Supratherapeutic dosing (over-dosing) was defined as a PCD higher than the CCD, indicating administration of iron in excess of the SmPC-recommended dose.

Financial Impact Assessment

Financial impact was assessed from two perspectives: direct medication costs and projected costs of clinical complications associated with inadequate iron repletion.

- Direct Medication Costs (False Cost Savings): Under-dispensing was defined as dispensing fewer vials than required to fulfill the CCD. The number of under-dispensed vials was calculated as: Expected vials (based on CCD ÷ 500 mg per vial, rounded up) minus Actual vials dispensed. The financial gap was calculated by multiplying under-dispensed vials by the institutional acquisition cost per 500 mg vial (356.35 SAR).
- Projected Complication Costs: Financial impact of subtherapeutic dosing was estimated for the pregnant subgroup where published outcome data were available. Using odds ratios from Detlefs et al. for inadequate response to iron therapy (preeclampsia OR = 1.54; preterm birth OR = 1.44), adjusted probabilities were calculated using the formula: $Adjusted probability=\frac{(OR \times baseline probability)}{[1 + (OR \times baseline probability)]}$ [4]

Baseline probabilities were derived from Saudi population data (preeclampsia 5%; preterm birth 10%)[5, 6]. Expected cases were calculated by multiplying adjusted probabilities by the number of under-dosed pregnant women. Treatment costs were estimated from published literature: $9,389 additional costs for preeclampsia management [7], and 60,000 SAR ($16,000) per premature infant for neonatal care in Saudi Arabia[8]. Complication costs for non-pregnant patients could not be quantified due to lack of published outcome-specific cost data; however, literature suggests inadequately treated anemia is associated with increased healthcare utilization and costs⁴².

The financial impact projections were based on the following assumptions: (1) Odds ratios for adverse outcomes associated with inadequate iron therapy response were derived from Detlefs et al., a large retrospective cohort study of pregnant women with iron deficiency anemia in the United States (preeclampsia OR = 1.54, 95% CI: 1.21–1.96; preterm birth OR = 1.44, 95% CI: 1.18–1.76)[4]; (2) These odds ratios were assumed to be generalizable to the Saudi pregnant population, acknowledging potential differences in baseline risk factors, healthcare access, and obstetric practices between populations; (3) Baseline probabilities for preeclampsia (5%) [5] and preterm birth (10%) [6]were derived from published Saudi epidemiological data; (4) All pregnant women receiving subtherapeutic FCM doses were assumed to have inadequate iron therapy response, which may overestimate complication rates; (5) Treatment costs were obtained from published literature [7, 8]and institutional data, and may not reflect current or facility-specific costs; and (6) Indirect costs such as productivity loss, long-term neonatal complications, and maternal psychological burden were not included. These assumptions introduce uncertainty into the financial projections, which should be interpreted as illustrative estimates rather than precise predictions.

**References**

[1]	Treatment of iron deficiency and iron deficiency anemia in adults - UpToDate, https://www.uptodate.com/contents/treatment-of-iron-deficiency-and-iron-deficiency-anemia-in-adults?sectionName=Oral%20iron&topicRef=144555&anchor=H5&source=bqp#H5 (accessed January 13, 2026).

[2]	Pavord S, Daru J, Prasannan N, et al. UK guidelines on the management of iron deficiency in pregnancy. *Br J Haematol* 2020; 188: 819–830.

[3]	Ferinject 50 mg iron/mL dispersion for injection/infusion. - Summary of Product Characteristics (SmPC) - (emc) | 5910, https://www.medicines.org.uk/emc/product/5910/smpc#gref (accessed January 13, 2026).

[4]	Detlefs SE, Jochum MD, Salmanian B, et al. The impact of response to iron therapy on maternal and neonatal outcomes among pregnant women with anemia. *Am J Obstet Gynecol MFM*; 4. Epub ahead of print March 1, 2022. DOI: 10.1016/J.AJOGMF.2022.100569.

[5]	Al-Rubaie ZTA, Hudson HM, Jenkins G, et al. Prediction of pre-eclampsia in nulliparous women using routinely collected maternal characteristics: a model development and validation study. *BMC Pregnancy Childbirth*; 20. Epub ahead of print January 6, 2020. DOI: 10.1186/S12884-019-2712-X.

[6]	Almaghaslah EH, Ibrahim I Al, Al-Zahir SS, et al. Prevalence of Preterm Birth in Saudi Arabia: A Systematic Review and Meta-Analysis. *Cureus* 2024; 16: e74562.

[7]	Li R, Kuklina E V., Ailes EC, et al. Medical expenditures for hypertensive disorders during pregnancy that resulted in a live birth among privately insured women. *Pregnancy Hypertens* 2021; 23: 155–162.

[8]	Saudi Arabia delivers ‘early warning’ on preterm births | Arab News, https://www.arabnews.com/node/1587341/saudi-arabia (accessed January 19, 2026).
