# Supplementary material for: Prescribing and dosing patterns of ferric carboxymaltose for iron deficiency anemia: a real-world study from Saudi Arabia
Source: Front Med (Lausanne). 2026 Aug 7;13:1864516. doi: 10.3389/fmed.2026.1864516 (PMC13493227; doi:10.3389/fmed.2026.1864516)
Supplement: Supplementary file 2 [file Table_2.DOCX]

**Supplementary Table S1**

**Ferric Carboxymaltose Dosing Table per Summary of Product Characteristics**

| **Hemoglobin Level** | **<35 kg** | **35 to <70 kg** | **≥70 kg** |
| --- | --- | --- | --- |
| <10 g/dL | 30 mg/kg | 1,500 mg | 2,000 mg |
| 10 to <14 g/dL | 15 mg/kg | 1,000 mg | 1,500 mg |
| ≥14 g/dL | 15 mg/kg | 500 mg | 500 mg |

*Maximum single dose: 1,000 mg or 20 mg/kg (infusion) / 15 mg/kg (injection). Minimum interval between doses: 7 days. Source: Ferinject Summary of Product Characteristics.³⁷*
